# Redox-active, luminescent coordination nanosheet capsules containing magnetite

**DOI:** 10.1038/s41598-020-70715-6

**Published:** 2020-08-14

**Authors:** Ryo Arai, Mengjuan Li, Ryojun Toyoda, Hiroaki Maeda, Hiroshi Nishihara

**Affiliations:** 1 Department of Chemistry, School of Science, The University of Tokyo, 7-3-1 Hongo, Bunkyo-ku, Tokyo, 113-0033 Japan; 2 College of Textile Science and Engineering, Jiangnan University, Wuxi, Jiangsu China; 3 Research Center for Science and Technology, Tokyo University of Science, 2641 Yamazaki, Noda, Chiba 278-8510 Japan

**Keywords:** Coordination chemistry, Metal-organic frameworks

## Abstract

Two-dimensional coordination nanosheets (CONASHs) are grown at the spherical liquid–liquid interface of a dichloromethane droplet in water to form zero-dimensional nano- and micro-capsules using a simple dropping method, a syringe-pump method, and an emulsion method. Reaction of 1,3,5-tris[4-(4′-2,2′:6′,2″-terpyridyl)phenyl]benzene (1) with Fe(BF_4_)_2_ affords electrochromic Fe(tpy)_2_ CONASH capsules and that of ligand 1 with ZnSO_4_ does photoluminescent Zn_2_(μ-O_2_SO_2_)_2_(tpy)_2_ CONASH capsules. Fe(tpy)_2_ CONASH capsules containing magnetite particles were produced by the syringe-pump method by adding magnetite to the aqueous phase, with the assembly and dispersion of the magnetite-containing CONASH capsules being easily controlled with a magnet. This indicates that physicochemically functional CONASH capsules are suitable for incorporating other functional materials to develop hybrid systems.

## Introduction

Two-dimensional (2D) materials such as graphene^1,2^ and transition-metal dichalcogenides^3,4^, have unique properties and functions for various applications^5–8^. Coordination nanosheets (CONASHs) are 2D materials that consist of metal ions and organic π-ligands^9,10^. Since the discovery of an electronically conducting bis(dithiolato)nickel nanosheet^11^, a range of CONASHs have been synthesized and various physical properties such as electronic conductivity^12,13^, energy storage^14^, redox activity^15^, luminescence^16^, photoelectric conversion^17^, and electrocatalytic activity^18^ have been enhanced by tuning the chemical structures. Films can be directly synthesized by a reaction at the liquid–liquid or gas–liquid interface, where metal ions and organic ligands exist in different phases. Most previous work on CONASHs has used planar interfaces to obtain planar films; however, other spherical functional nanomaterials have been extensively studied^19–31^.

In this study, we used the spherical liquid–liquid interface of droplets to synthesize spherical CONASHs focusing on their functions. Using 1,3,5-tris[4-(4′-2,2′:6′,2″-terpyridyl)phenyl]benzene (**1**), which is a three-way bridging tris(terpyridine) ligand, we obtained electrochromic Fe(tpy)_2_^32–34^ CONASH capsules and photoluminescent Zn_2_(SO_4_)_2_(tpy)_2_^16,35^ CONASH capsules by a simple dropping method, a syringe pump method and an emulsion method. The formation of the CONASHs at the liquid–liquid interfaces is accelerated by the addition of surfactant such as sodium dodecyl sulfate (SDS), allowing the CONASH capsules to be formed within 15 min^36,37^. The surfactant also stabilizes the droplets during synthesis^38,39^. We also encapsulated magnetite (Fe_3_O_4_) particles^40^ in the Fe(tpy)_2_ shell, and the magnetite-containing Fe(tpy)_2_ CONASH capsules could be controlled with a magnet. Our results show that physicochemically functional CONASH capsules are suitable for incorporating other functional materials to develop hybrid systems.

### Synthesis of electrochromic Fe(tpy)_2_ CONASH capsules using the dropping method

**Figure 1:**
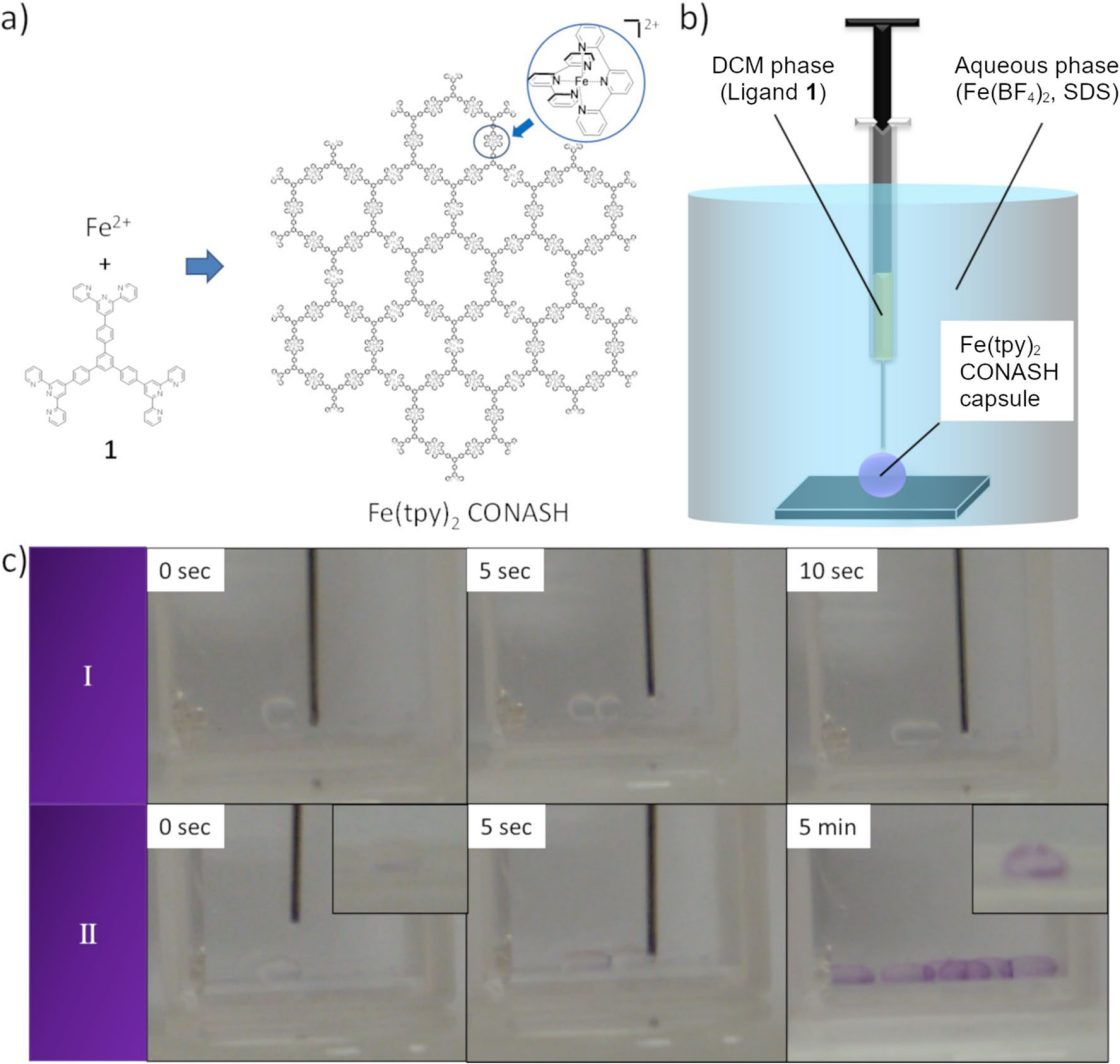
Dropping method to form Fe(tpy)_2_ CONASH capsules. (**a**) Chemical structure of Fe(tpy)_2_ CONASH. (**b**) Conceptual image of dropping method. (**c**) Snapshots of Fe(tpy)_2_ CONASH capsules fabricated by dropping method, (I) in the absence of surfactant and (II) in the presence of SDS.

We previously synthesized a planar Fe(tpy)_2_ CONASH film (Fig. 1a) using the coordination reaction at the planar interface between Fe(BF_4_)_2_ in water and the tris(terpyridine) ligand, **1**. The reaction was sluggish and took several hours to grow a film of sub-micron thickness. The reaction to fabricate CONASH capsules occurs at the spherical liquid–liquid interface between an aqueous phase containing metal ions and an organic phase containing organic ligands (Fig. 1b). Because the reaction is slow, the droplets easily fuse (Fig. 1c I). Adding a surfactant, such as SDS, accelerated the coordination reaction at the interface, shortening the reaction time to 15 min (Fig. 1c II). To grow the Fe(tpy)_2_ capsules with a shell thickness of 20 nm (Supplementary Fig. S1), droplets of ligand **1** in dichloromethane (DCM) were created in an aqueous solution of Fe(BF_4_)_2_. Due to the fast reaction, Fe(tpy)_2_ capsules were fabricated without the droplets fusing. Amphiphilic SDS makes a metal complex with a ferrous ion in an aqueous phase. The formed complex easily moves to organic phase and works as an iron ion source of Fe(tpy)_2_ complex. Hence, SDS can work as an accelerator of the complexation reaction of Fe(tpy)_2_ complex at the organic-water solution interface.

**Figure 2:**
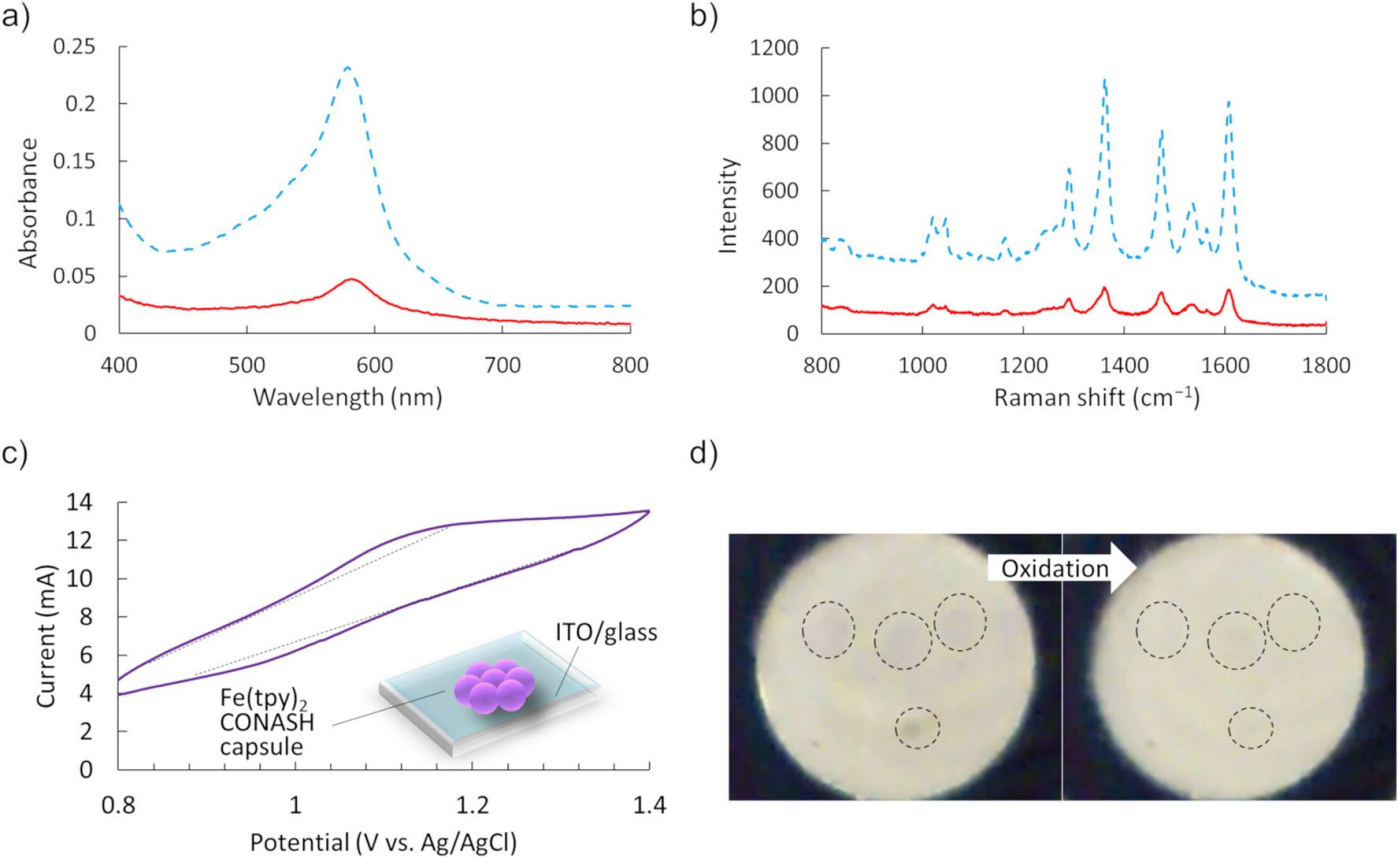
Electrochemical and spectral properties. (**a**) UV–Vis absorption spectra of Fe(tpy)_2_ CONASH capsules (solid line) and a CONAH film (broken line). (**b**) Raman spectra of Fe(tpy)_2_ CONASH capsules (solid line) and a CONASH film (broken line). (**c**)  Cyclic voltammogram of Fe(tpy)_2_ CONASH capsules and schematic of the capsules on an ITO electrode. (**d**) Images of Fe(tpy)_2_ CONASH capsules oxidized electrochemically. (**b**) Cyclic voltammogram of Fe(tpy)_2_ CONASH capsules and schematic of the capsules on an ITO electrode.

The characterization of the Fe(tpy)_2_ capsules was conducted using UV–Vis and Raman spectra. The capsule gives a peak derived from MLCT of Fe(tpy)_2_ complex at 580 nm corresponding with that of Fe(tpy)_2_ nanosheet (Fig. 2a). In addition, Raman spectra of Fe(tpy)_2_ capsule and nanosheet show good agreement each other (Fig. 2b). These spectroscopic results suggest that the capsule is composed of Fe(tpy)_2_ nanosheet which was formed at the spherical liquid–liquid interface. In the simple dropping method using a micro-syringe, the place and size of the spherical capsules in the size range 1–2.5 mm were easily controlled (Supplementary Fig. S2).

Cyclic voltammetry of a single Fe(tpy)_2_ CONASH capsule 0.95 mm in diameter on an indium–tin oxide (ITO) glass plate was performed in 0.1 M Na_2_SO_4_ aq (Fig. 2c, Supplementary Fig. S3). Small oxidation and re-reduction peaks derived from the redox reaction of [Fe(tpy)_2_]^3+/2+^ appeared at 1.10 and 0.98 V (vs. Ag/AgCl) and thus *E*^0′^ = 1.04 V (vs. Ag/AgCl) with a peak-to-peak potential of 120 mV, indicating a chemically reversible, slow electron-transfer process, probably because of the weak physical contact between the droplet and ITO.

We previously reported that the planar Fe(tpy)_2_ CONASH film on an ITO electrode undergoes rapid and durable electrochromism^34^; the color changed reversibly between purple and pale yellow for [Fe(tpy)_2_]^2+^ and [Fe(tpy)_2_]^3+^, respectively. In the present study, we observed a similar color change (Fig. 2d), although the color was faint because the Fe(tpy)_2_ shell was thin. The color of the whole shell changed, even though the contact area of the CONASH capsule with the electrode was very small indicating rapid electron hopping between Fe(tpy)_2_ sites through the molecular framework of the shell.

### Synthesis of Fe(tpy)_2_ CONASH capsules using the syringe-pump method and encapsulation of magnetite particles

**Figure 3:**
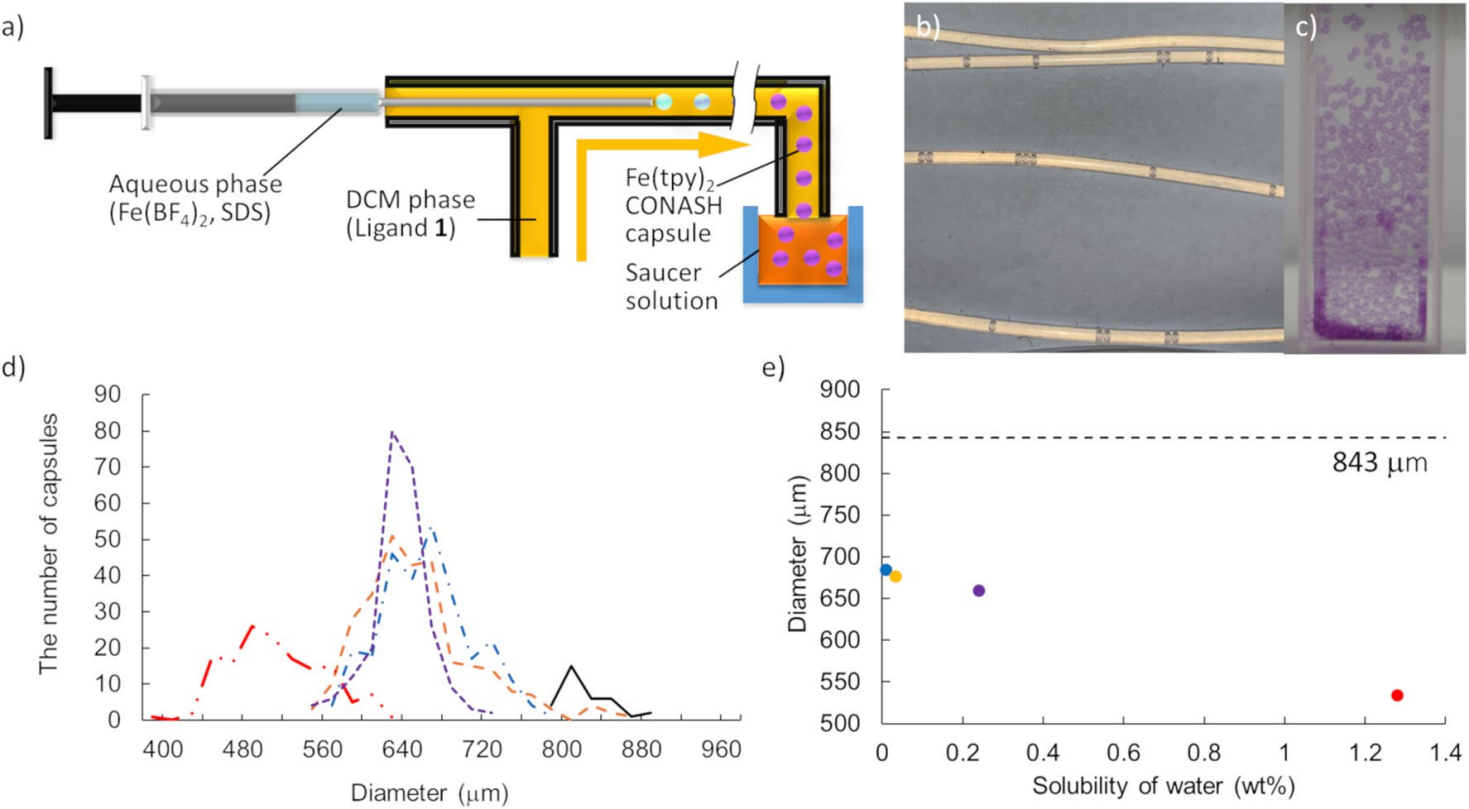
Syringe-pump method to form Fe(tpy)_2_ CONASH capsules. (**a**) Schematic of the syringe-pump method. (**b**) Photograph of Fe(tpy)_2_ CONASH capsules during the formation in tubes. (**c**) Photograph of Fe(tpy)_2_ CONASH capsules in hexane. (**d**) Size distribution of Fe(tpy)_2_ CONASH capsules in the tube (solid line) and saucer organic solvents; diethyl ether (two-dot chain line), DCM (dotted line), toluene (broken line), and hexane (chain line). (**e**) Diameter of Fe(tpy)_2_ CONASH capsules vs. solubility of water in a given organic solvent; diethyl ether (red), DCM (purple), toluene (orange), and hexane (blue). The diameter in the tube, 843.2 μm is given for comparison.

The syringe-pump method, in which water droplets are formed and flow continuously in the moving organic solution, was used to obtain Fe(tpy)_2_ CONASH capsules with a narrow size distribution, easily and constantly (Fig. 3a). The size of the droplets depended on the radius of tubes^41,42^, while the flow rate and tube length controlled the reaction time at the liquid–liquid interface, and thus the thickness of the Fe(tpy)_2_ shells (Fig. 3b). Fe(tpy)_2_ CONASH capsules were collected at the end of the tube in a saucer solution (Fig. 3a,c). We synthesized Fe(tpy)_2_ CONASH capsules using DCM as the organic phase of the reaction. Capsules with a diameter of 843 ± 28 μm and a shell thickness of 250 nm were obtained under typical conditions involving 25 mM Fe(BF_4_)_2_; 10 mM SDS; aqueous solution flow rate of 0.5 mL h^−1^; syringe inner diameter 0.15 mm; 60 μM ligand **1**; DCM solution flow rate 10 mL h^–1^, and tube inner diameter 0.86 mm (Supplementary Fig. S4). Capsule size depended on the saucer solution, and was 659 ± 29 μm in DCM, 684 ± 44 μm in hexane, 677 ± 57 μm in toluene, and 534 ± 48 μm in diethyl ether (Fig. 3d,e, Supplementary Fig. S5). The diameter of Fe(tpy)_2_ CONASH capsules was thus expected to depend on the solubility of water in the saucer solvent; larger in hydrophobic solvents than in hydrophilic solvents. In hydrophilic solvents, water dissolved in the outer organic solvents and was transferred out of the Fe(tpy)_2_ shell. This dependency of capsule size on the outer environment suggests that the capsules might have the permeability of water due to the porous structures of Fe(tpy)_2_ CONASH.

**Figure 4:**
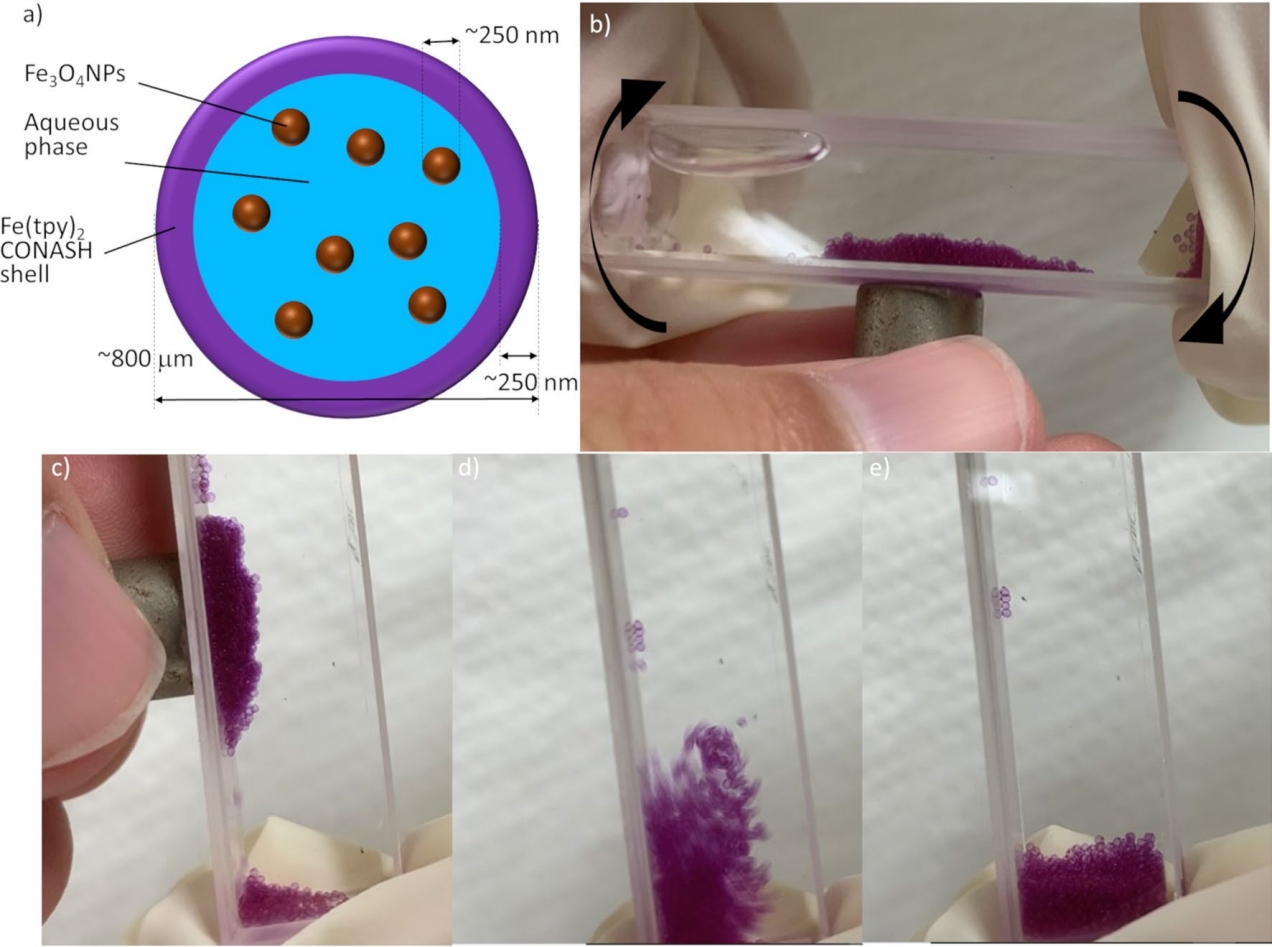
Magnetite-containing Fe(tpy)_2_ CONASH capsules. (**a**) Schematic of magnetite-containing Fe(tpy)_2_ CONASH capsules. (**b–e**) Snapshots of the magnetite-containing Fe(tpy)_2_ CONASH capsules attracted by a magnet.

The space in Fe(tpy)_2_ CONASH capsules could be used to add further functionality. Magnetic particles and beads have been used for various applications in fields such as biomedical engineering and heterogeneous catalysis because their aggregation and dispersion are easily controlled by an external magnetic field. We introduced magnetite (Fe_3_O_4_) particles into CONASH capsules by a simple syringe-pump method. Magnetite particles with a diameter of 250 nm^40^ were dispersed in an aqueous solution of Fe(BF_4_)_2_. The Fe(tpy)_2_ CONASH capsules emerged at the water–DCM interface and contained the magnetite particles (Fig. 4a). This method of assembly of CONASH capsules could be controlled externally. The magnetite-containing CONASH capsules were strongly attracted by magnets (Fig. 4b–e), demonstrating that the Fe(tpy)_2_ CONASH capsules showed the functionality of the original Fe(tpy)_2_ CONASH shell and the incorporated magnetite.

### Synthesis of photoluminescent Zn_2_(SO_4_)_2_(tpy)_2_ CONASH capsules

**Figure 5:**
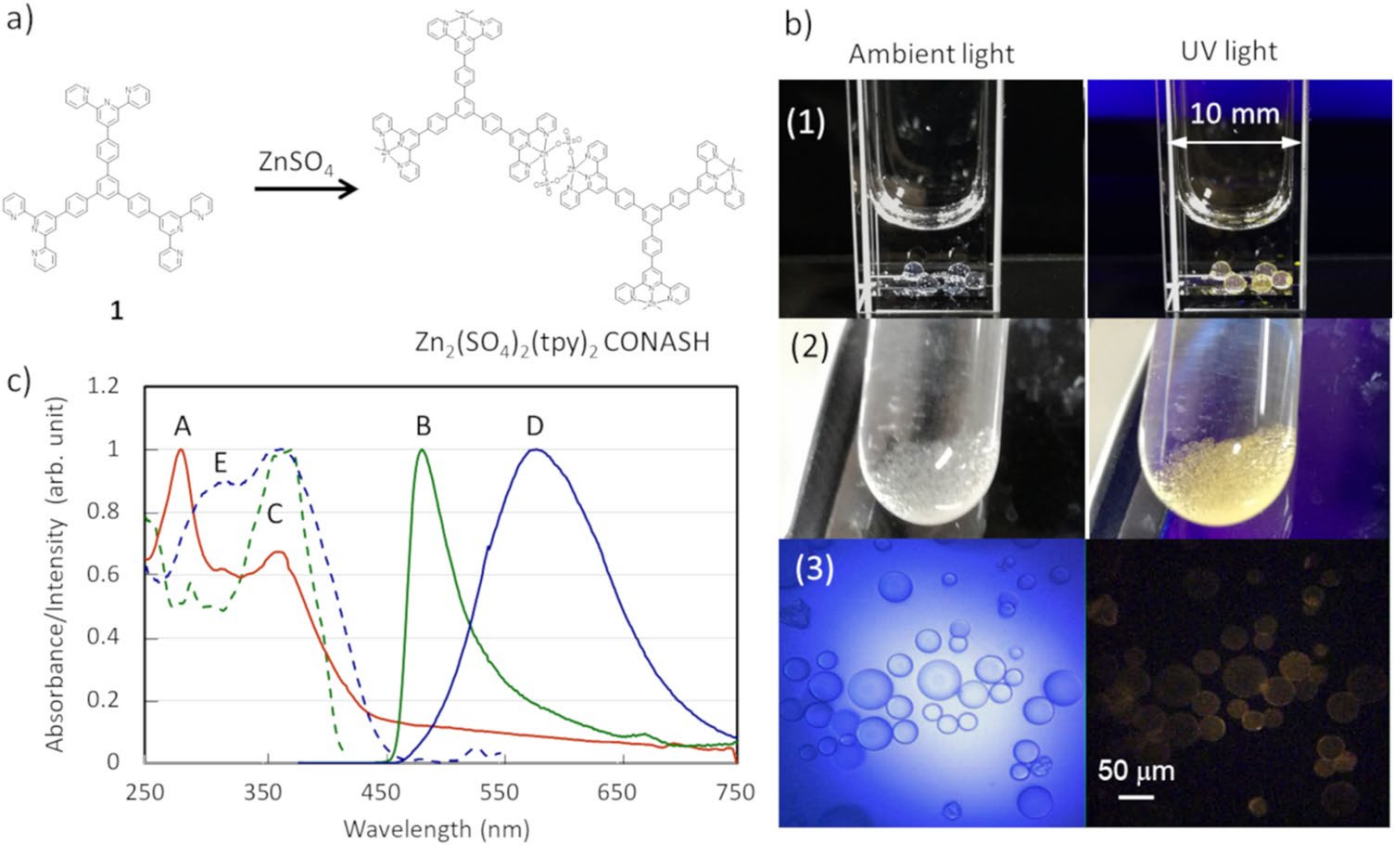
Zn_2_(SO_4_)_2_(tpy)_2_ CONASH capsules. (**a**) Synthesis of Zn_2_(SO_4_)_2_(tpy)_2_ CONASH capsules. (**b**) Photographs and microscope images of Zn_2_(SO_4_)_2_(tpy)_2_ CONASH capsules obtained by the dropping method (1) and by the emulsion method (2, 3) under ambient and 365 nm UV light. (**c**) Absorption (A), emission (B) and excitation (C) spectra of Zn_2_(SO_4_)_2_(tpy)_2_ CONASH capsules in DCM, and emission (D) and excitation (E) spectra of Zn_2_(SO_4_)_2_(tpy)_2_ CONASH capsules in water.

We previously reported that the liquid–liquid interfacial coordination reaction of a DCM solution of ligand **1** and an aqueous solution of ZnSO_4_ produces a photoluminescent sulfate-bridged dimeric zinc terpyridine complex polymer film called Zn_2_(SO_4_)_2_(tpy)_2_ CONASH (Fig. 5a)^16^. Therefore, we investigated the synthesis of Zn_2_(SO_4_)_2_(tpy)_2_ CONASH capsules using not only the dropping method but also the emulsion method, in which a DCM solution of ligand **1** and an aqueous solution of ZnSO_4_ (1:3 v/v) were shaken together vigorously in a test tube to obtain an emulsion. The emulsion interface was left to stand for 1 h, and Zn_2_(SO_4_)_2_(tpy)_2_ CONASH formed at the interface as a transparent film (Fig. 5b). Note that in the case of the emulsion method, shaking a mixture of the dichloromethane solution and the aqueous solution in a test tube makes smaller microcapsules than those produced by the other methods. We think they have sufficiently large surface area so that the interfacial reaction proceeds rapidly even without adding SDS. The Zn_2_(SO_4_)_2_(tpy)_2_ CONASH capsules were 20–50 μm in size, showed UV absorption bands at 372 and 281 nm in DCM and exhibited solvatoluminochromism^16^; pale yellow-green photoluminescence with an emission maximum at 580 nm in water and pale blue photoluminescence with an emission maximum at 484 nm in DCM upon UV irradiation (365 nm) (Fig. 5c).

## Conclusions

We produced CONASH capsules from 2D coordination polymers using the spherical liquid–liquid interface of DCM droplets in water. The simple dropping method, the syringe-pump method, and the emulsion method were used to synthesize Fe(tpy)_2_ CONASH capsules and Zn_2_(SO_4_)_2_(tpy)_2_ CONASH capsules. The Fe(tpy)_2_ CONASH capsules retained the electrochromism of the nanosheet and the Zn_2_(SO_4_)_2_(tpy)_2_ CONASH capsules retained the photoluminescence. The syringe-pump method incorporated magnetite into the Fe(tpy)_2_ CONASH capsules by mixing magnetite in the aqueous phase. The assembly and dispersion of the capsules could be controlled by a magnet. Our results demonstrate that the application range of CONASHs can be expanded by using a zero-dimensional material instead of a 2D material.

## Methods

### Synthesis of Fe(tpy)_2_ CONASH capsules by the dropping method

A DCM solution of ligand **1**^33, 43^ (60 μM) and an aqueous solution containing 25 mM Fe(BF_4_)_2_ and saturated SDS were prepared. The aqueous solution (10 mL) was poured into a vial. A droplet of DCM solution (1 μL) was placed in the aqueous solution by slow pipetting. After 15 min, the purple Fe(tpy)_2_ CONASH sphere emerged at the interface. Some measurements were conducted in situ or the sphere was removed with the substrate and washed with deionized water, ethanol, and DCM.

### Synthesis of Fe(tpy)_2_ CONASH capsules by the syringe pump method

A DCM solution of the ligand **1** (60 μM) and an aqueous solution containing 25 mM Fe(BF_4_)_2_ and saturated SDS were prepared. The aqueous phase was injected through a hollow needle (25 s gauge, 90° tip, inner diameter of 0.15 mm, outer diameter at the tip of 0.51 mm) in a co-floating stream of the DCM phase floating through polytetrafluoroethylene tubing (inner diameter 0.86 mm). The Fe(tpy)_2_ CONASH capsules were collected and kept in organic solvent. The aqueous and DCM phases were both supplied by syringe pump set at 0.5 and 10 mL h^−1^, respectively.

### Synthesis of Zn_2_(SO_4_)_2_(tpy)_2_ CONASH capsules by the emulsion method

A DCM solution of ligand **1** (0.1 mM) and an aqueous solution of ZnSO_4_ (5.0 mM) were used to synthesize Zn_2_(SO_4_)_2_(tpy)_2_ CONASH capsules by the liquid–liquid interfacial coordination reaction. The DCM solution and aqueous solution (1:3 v/v) were injected into a test tube and mixed by vigorous shaking at room temperature. The emulsion interface was allowed to stand for 1 h, and the Zn_2_(SO_4_)_2_(tpy)_2_ CONASH film generated at the interfaces of the emulsion and Zn_2_(SO_4_)_2_(tpy)_2_ CONASH droplets emerged as light-yellow spheres.

## Supplementary information

Supplementary Information

## Data Availability

The data for the plots in this work and other findings from this study are available from the corresponding author upon reasonable request.

## References

[1] Han W, Kawakami RK, Gmitra M, Fabian J (2014). Graphene spintronics. Nat. Nanotechnol..

[2] Li L, Qin R, Li H, Yu L, Liu Q, Luo G, Gao Z, Lu J (2011). Functionalized graphene for high-performance two-dimensional spintronics devices. ACS Nano.

[3] Eda G, Yamaguchi H, Voiry D, Fujita T, Chen M, Chhowalla M (2011). Photoluminescence from chemically exfoliated MoS_2_. Nano Lett..

[4] Mak KF, Shan J (2016). Photonics and optoelectronics of 2D semiconductor transition metal dichalcogenides. Nat. Photonics.

[5] Zhang H (2015). Ultrathin two-dimensional nanomaterials. ACS Nano.

[6] Bauer T, Zheng Z, Renn A, Enning R, Stemmer A, Sakamoto J, Schlüter D (2011). Synthesis of free-standing, monolayered organometallic sheets at the air/water interface. Angew. Chem. Int. Ed..

[7] Cassabois G, Valvin P, Gil B (2016). Hexagonal boron nitride is an indirect bandgap semiconductor. Nat. Photonics.

[8] Burch KS, Mandrus D, Park JG (2018). Magnetism in two-dimensional van der Waals materials. Nature.

[9] Sakamoto R, Takada K, Pal T, Maeda H, Kambe T, Nishihara H (2017). Coordination nanosheets (CONASHs): Strategies, structures and functions. Chem. Commun..

[10] Sakamoto R, Takada K, Sun X, Pal T, Tsukamoto T, Phua EJH, Rapakousiou A, Hoshiko K, Nishihara H (2016). The coordination nanosheet (CONASH). Coord. Chem. Rev..

[11] Kambe T, Sakamoto R, Kusamoto T, Pal T, Fukui N, Shimojima T, Wang Z, Hirahara T, Ishizaka K, Hasegawa S, Liu F, Nishihara H (2014). Redox control and high conductivity of nickel bis(dithiolene) complex π-nanosheet: A potential organic two-dimensional topological insulator. J. Am. Chem. Soc..

[12] Sun X, Wu K-H, Sakamoto R, Kusamoto T, Maeda H, Nishihara H (2017). Conducting π-conjugated bis(iminothiolato)nickel nanosheet. Chem. Lett..

[13] Pal T, Kambe T, Kusamoto T, Foo ML, Matsuoka R, Sakamoto R, Nishihara H (2015). Interfacial synthesis of electrically conducting palladium bis(dithiolene) complex nanosheet. ChemPlusChem.

[14] Wada K, Sakaushi K, Sasaki S, Nishihara H (2018). Multielectron-transfer-based rechargeable energy storage of two-dimensional coordination frameworks with non-innocent ligands. Angew. Chem. Int. Ed..

[15] Phua EJH, Wu K-H, Wada K, Kusamoto T, Maeda H, Cao J, Sakamoto R, Masunaga H, Sasaki S, Mei J-W, Jiang W, Liu F, Nishihara H (2018). Oxidation-promoted interfacial synthesis of redox-active bis(diimino)nickel nanosheet. Chem. Lett..

[16] Tsukamoto T, Takada K, Sakamoto R, Matsuoka R, Toyoda R, Maeda H, Yagi T, Nishikawa M, Shinjo N, Amano S, Iokawa T, Ishibashi N, Oi T, Kanayama K, Kinugawa R, Koda Y, Komura T, Nakajima S, Fukuyama R, Fuse N, Mizui M, Miyasaki M, Yamashita Y, Yamada K, Zhang W, Han R, Liu W, Tsubomura T, Nishihara H (2017). Coordination nanosheets based on terpyridine-zinc(II) complexes: As photoactive host materials. J. Am. Chem. Soc..

[17] Sakamoto R, Yagi T, Hoshiko K, Kusaka S, Matsuoka R, Maeda H, Liu Z, Liu Q, Wong W-Y, Nishihara H (2017). Photofunctionality in porphyrin-hybridized bis(dipyrrinato)zinc(II) complex micro- and nanosheets. Angew. Chem. Int. Ed..

[18] Sun X, Wu K-H, Sakamoto R, Kusamoto T, Maeda H, Ni X, Jiang W, Liu F, Sasaki S, Masunaga H, Nishihara H (2017). Bis(aminothiolato)nickel nanosheet as a redox switch for conductivity and an electrocatalyst for the hydrogen evolution reaction. Chem. Sci..

[19] Zhou D, Liu R, He Y-B, Li F, Liu M, Li B, Yang Q-H, Cai Q, Kang F (2016). SiO_2_ hollow nanosphere-based composite solid electrolyte for lithium metal batteries to suppress lithium dendrite growth and enhance cycle life. Adv. Energy Mater..

[20] Jeong GY, Ricco R, Liang K, Ludwig J, Kim J-O, Falcaro P, Kim D-P (2015). Bioactive MIL-88A framework hollow spheres via interfacial reaction in-droplet microfluidics for enzyme and nanoparticle encapsulation. Chem. Mater..

[21] Guan C, Sumboja A, Wu H, Ren W, Liu X, Zhang H, Liu Z, Cheng C, Pennycook SJ, Wang J (2017). Hollow Co_3_O_4_ nanosphere embedded in carbon arrays for stable and flexible solid-state zinc-air batteries. Adv. Mater..

[22] Xia XH, Tu JP, Wang XL, Gu CD, Zhao XB (2011). Mesoporous Co_3_O_4_ monolayer hollow-sphere array as electrochemical pseudocapacitor material. Chem. Commun..

[23] Furukawa S, Reboul J, Diring S, Sumida K, Kitagawa S (2014). Structuring of metal-organic frameworks at the mesoscopic/macroscopic scale. Chem. Soc. Rev..

[24] Liang Z, Yang Z, Yuan H, Wang C, Qi J, Liu K, Cao R, Zheng H (2018). A protein@metal–organic framework nanocomposite for pH-triggered anticancer drug delivery. Dalton Trans..

[25] Xu Z, Zhang J, Pan T, Li H, Huo F, Zheng B, Zhang W (2020). Encapsulation of hydrophobic guests within metal−organic framework capsules for regulating host−guest interaction. Chem. Mater..

[26] Kandambeth S, Venkatesh V, Shinde DB, Kumari S, Halder A, Verma S, Banerjee R (2015). Self-templated chemically stable hollow spherical covalent organic framework. Nat. Commun..

[27] Lu Z, Liu Y, Liu X, Li S, Li Y, Yang S, Qin Y, Zheng L, Zhang H (2019). A hollow microshuttle-shaped capsule covalent organic framework for protein adsorption. J. Mater. Chem. B.

[28] Guo J, Ping Y, Ejima H, Alt K, Meissner M, Richardson JJ, Yan Y, Peter K, von Elverfeldt D, Hagemeyer CE, Caruso F (2014). Engineering multifunctional capsules through the assembly of metal-phenolic networks. Angew. Chem. Int. Ed..

[29] Ameloot R, Vermoortele F, Vanhove W, Roeffaers MBJ, Sels BF, De Vos DE (2011). Interfacial synthesis of hollow metal-organic framework capsules demonstrating selective permeability. Nat. Chem..

[30] Wu S, Xin Z, Zhao S, Sun S (2019). High-throughput droplet microfluidic synthesis of hierarchical metal-organic framework nanosheet microcapsules. Nano Res..

[31] Wang B, Prinsen P, Wang H, Bai Z, Wang H, Luque R, Xuan J (2017). Macroporous materials: Microfluidic fabrication, functionalization and applications. Chem. Soc. Rev..

[32] Mondal S, Yoshida T, Higuchi M (2019). Electrochromic devices using Fe(II)-based metallo-supramolecular polymer: Introduction of ionic liquid as electrolyte to enhance the thermal stability. J. Soc. Inf. Disp..

[33] Takada K, Sakamoto R, Yi S-T, Katagiri S, Kambe T, Nishihara H (2015). Electrochromic bis(terpyridine)metal complex nanosheets. J. Am. Chem. Soc..

[34] Maeda H, Sakamoto R, Nishihara H (2017). Interfacial synthesis of electrofunctional coordination nanowires and nanosheets of bis(terpyridine) complexes. Coord. Chem. Rev..

[35] Jiang T, Lu N, Hang Y, Yang J, Mei J, Wang J, Hua J, Tian H (2016). Dimethoxy triarylamine-derived terpyridine-zinc complex: A fluorescence light-up sensor for citrate detection based on aggregation-induced emission. J. Mater. Chem. C.

[36] Bode G, Lade M, Schomäcker R (2000). The kinetics of an interfacial reaction in microemulsions with excess phases. Chem. Eng. Technol..

[37] Piradashvili K, Alexandrino EM, Wurm FR, Landfester K (2016). Reactions and polymerizations at the liquid-liquid interface. Chem. Rev..

[38] Soma J, Papadopoulos KD (1996). Ostwald ripening in sodium dodecyl sulfate-stabilized decane-in-water emulsions. J. Colloid Interface Sci..

[39] Lin Y, Ng KM, Chan CM, Sun G, Wu J (2011). High-impact polystyrene/halloysite nanocomposites prepared by emulsion polymerization using sodium dodecyl sulfate as surfactant. J. Colloid Interface Sci..

[40] Liu J, Sun Z, Deng Y, Zou Y, Li C, Duo X, Xiong L, Gao Y, Li F, Zhao D (2009). Highly water-dispersible biocompatible magnetite particles with low cytotoxicity stabilized by citrate groups. Angew. Chem. Int. Ed..

[41] Christopher GF, Anna SL (2017). Microfluidic methods for generating continuous droplet streams. J. Phys. D. Appl. Phys..

[42] Garstecki P, Fuerstman MJ, Stone HA, Whitesides GM (2006). Formation of droplets and bubbles in a microfluidic T-junction—Scaling and mechanism of break-up. Lab Chip.

[43] Cavazzini M, Quici S, Scalera C, Puntoriero F, La Ganga G, Campagna S (2009). Synthesis, characterization, absorption spectra, and luminescence properties of multinuclear species made of Ru(II) and Ir(III) chromophores. Inorg. Chem..

