# Supplementary material for: Redox-active, luminescent coordination nanosheet capsules containing magnetite
Source: Sci Rep. 2020 Aug 14;10:13818. doi: 10.1038/s41598-020-70715-6 (PMC7429495; doi:10.1038/s41598-020-70715-6)
Supplement: Supplementary file 1 — Supplementary Information [file 41598_2020_70715_MOESM1_ESM.pdf]

SUPPLEMENTARY INFORMATION

Redox-active, luminescent coordination nanosheet capsules containing magnetite

Ryo Arai[1], Mengjuan Li[1,2], Ryojun Toyoda[1], Hiroaki Maeda[1,3], and Hiroshi Nishihara[1,3*]

[1] Department of Chemistry, School of Science, The University of Tokyo, 7-3-1 Hongo, Bunkyo-ku, Tokyo 113-0033, Japan. [2] College of Textile Science and Engineering, Jiangnan University, Wuxi, Jiangsu, China. [3] Research Center for Science and Technology, Tokyo University of Science, 2641 Yamazaki, Noda, Chiba, 278-8510 Japan. *e-mail nisihara@rs.tus.ac.jp

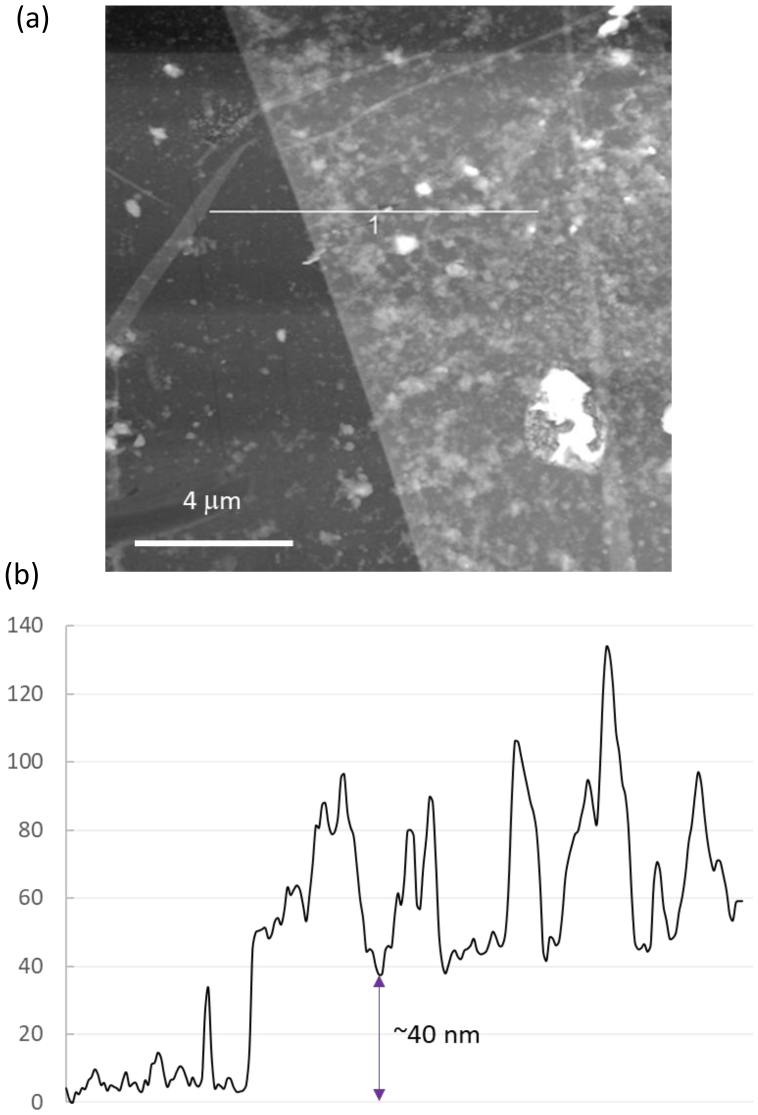

Supplementary Figure 1| AFM analysis of a $Fe(tpy)_2$ CONASH capsule fabricated by the dropping method. (a) A topological image. (b) The height profile along line 1.

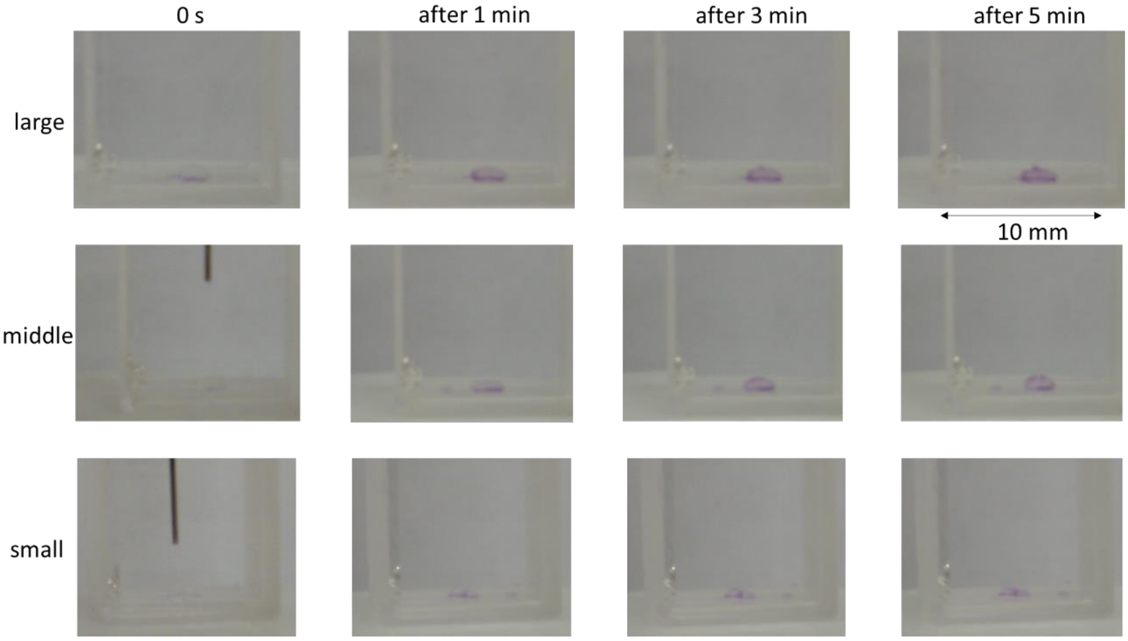

Supplementary Figure 2| Photographs of CONASH capsules formed after dropping a DCM solution of ligand **1** in an aqueous solution of $Fe(BF_4)_2$ and SDS.

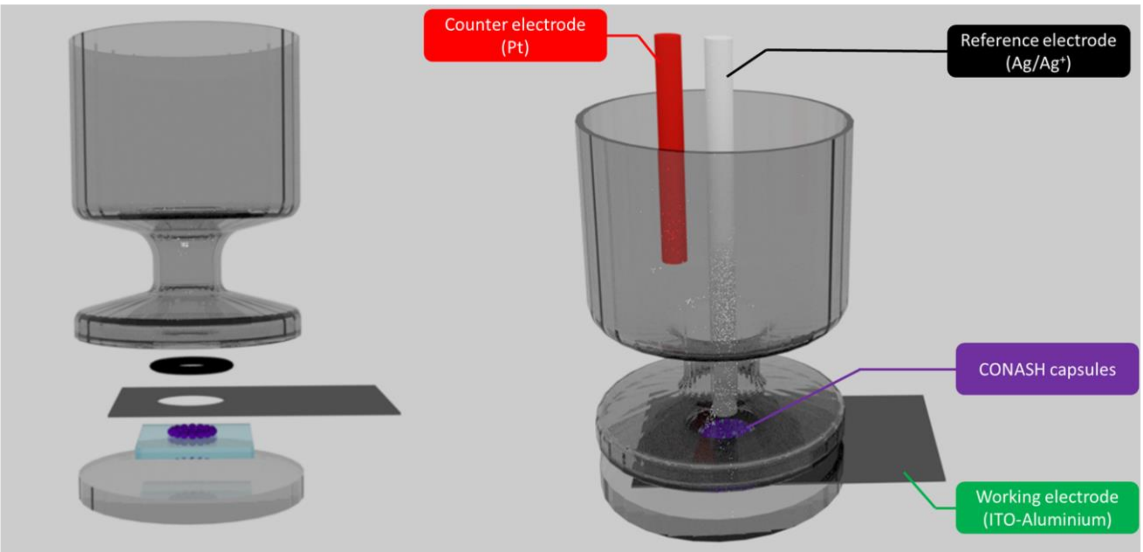

Supplementary Figure 3| Setup of electrochemical measurement of CONASH capsules.

(a)

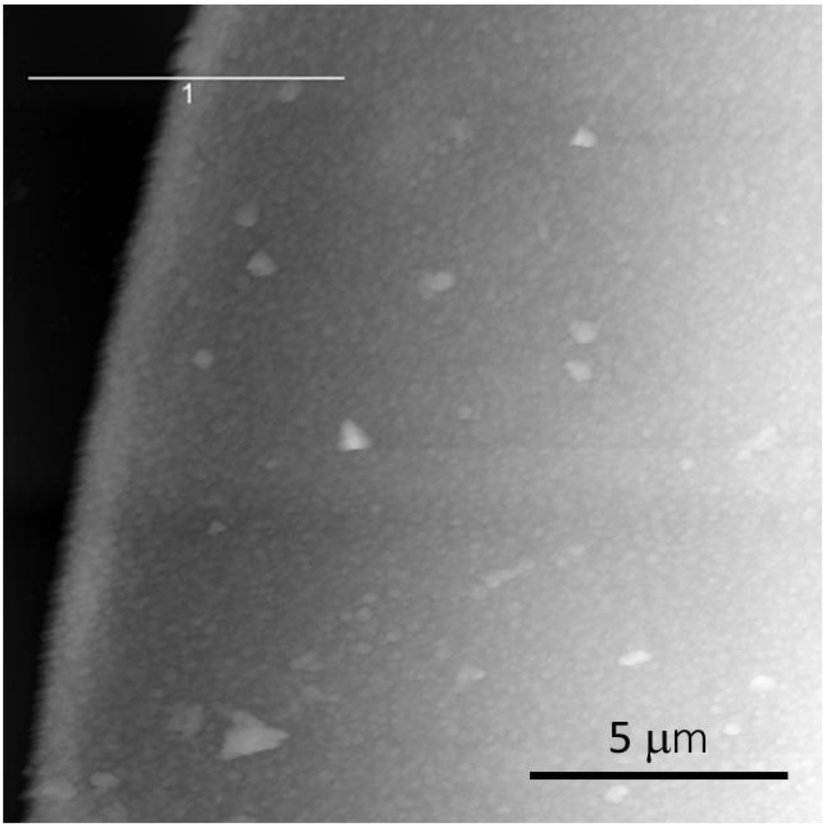

(b)

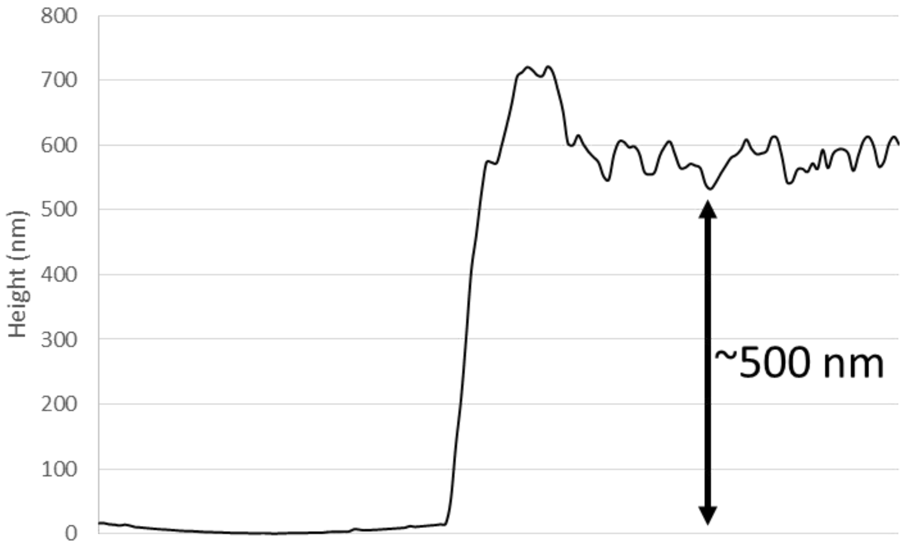

Supplementary Figure 4| AFM analysis of a $Fe(tpy)_2$ CONASH capsule fabricated by the syringe pump method. (a) A topological image. (b) The height profile along line 1.

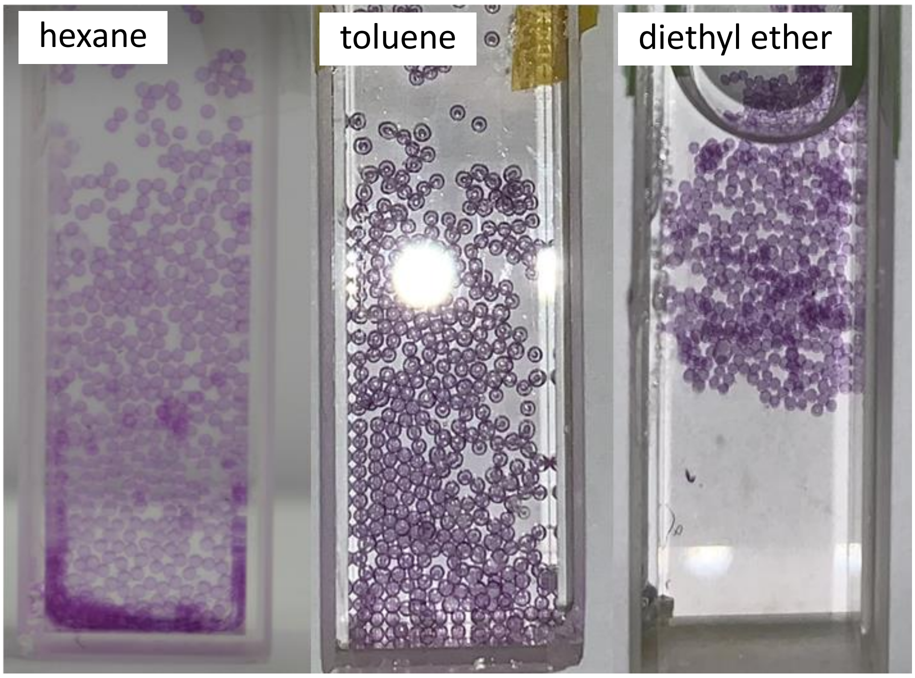

Supplementary Figure 5| Images of $Fe(tpy)_2$ CONASH capsules collected in hexane, toluene and diethyl ether.

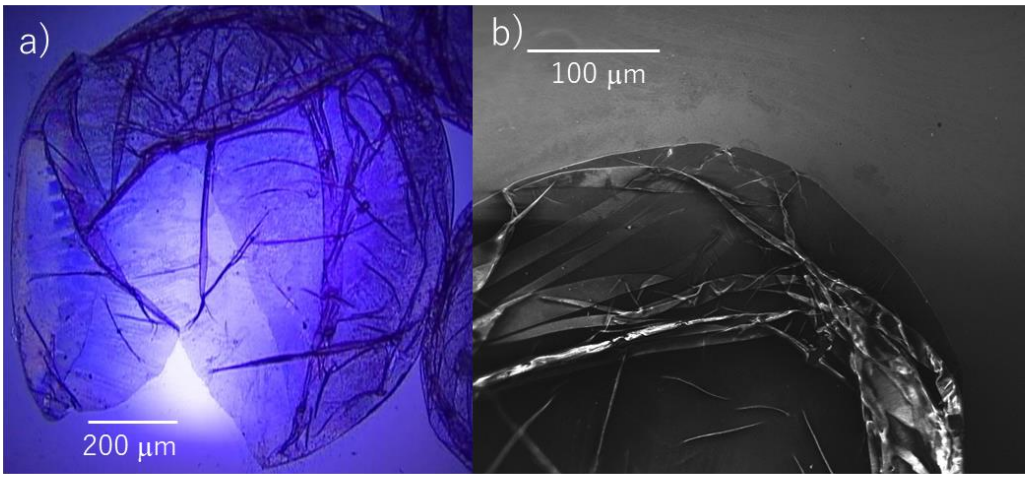

Supplementary Figure 6| An optical microscope image of a $Fe(tpy)_2$ CONASH capsule on a glass plate (a), and a SEM image of a $Fe(tpy)_2$ CONASH capsule on silicon fabricated by the syringe pump method (b).
